# Serum Levels of Acyl-Carnitines along the Continuum from Normal to Alzheimer's Dementia

**DOI:** 10.1371/journal.pone.0155694

**Published:** 2016-05-19

**Authors:** Adriana Cristofano, Nadia Sapere, Giancarlo La Marca, Antonella Angiolillo, Michela Vitale, Graziamaria Corbi, Giovanni Scapagnini, Mariano Intrieri, Claudio Russo, Gaetano Corso, Alfonso Di Costanzo

**Affiliations:** 1 Centre for Research and Training in Medicine for Aging, Department of Medicine and Health Sciences "Vincenzo Tiberio", University of Molise, Campobasso, Italy; 2 Newborn Screening, Biochemistry and Pharmacology Laboratories, Clinic of Pediatric Neurology, Meyer University Children’s Hospital, Florence, Italy; 3 Department of Neurosciences, Psychology, Pharmacology and Child Health, University of Florence, Florence, Italy; 4 Department of Clinical and Experimental Medicine, University of Foggia, Foggia, Italy; Biomedical Research Foundation, UNITED STATES

## Abstract

This study aimed to determine the serum levels of free L-carnitine, acetyl-L-carnitine and 34 acyl-L-carnitine in healthy subjects and in patients with or at risk of Alzheimer’s disease. Twenty-nine patients with probable Alzheimer’s disease, 18 with mild cognitive impairment of the amnestic type, 24 with subjective memory complaint and 46 healthy subjects were enrolled in the study, and the levels of carnitine and acyl-carnitines were measured by tandem mass spectrometry. The concentrations of acetyl-L-carnitine progressively decreased passing from healthy subjects group (mean±SD, 5.6±1.3 μmol/L) to subjective memory complaint (4.3±0.9 μmol/L), mild cognitive impairment (4.0±0.53 μmol/L), up to Alzheimer’s disease (3.5±0.6 μmol/L) group (p<0.001). The differences were significant for the comparisons: healthy subjects vs. subjective memory complaint, mild cognitive impairment or Alzheimer’s disease group; and subjective memory complaint vs. Alzheimer’s disease group. Other acyl-carnitines, such as malonyl-, 3-hydroxyisovaleryl-, hexenoyl-, decanoyl-, dodecanoyl-, dodecenoyl-, myristoyl-, tetradecenoyl-, hexadecenoyl-, stearoyl-, oleyl- and linoleyl-L-carnitine, showed a similar decreasing trend, passing from healthy subjects to patients at risk of or with Alzheimer’s disease. These results suggest that serum acetyl-L-carnitine and other acyl-L-carnitine levels decrease along the continuum from healthy subjects to subjective memory complaint and mild cognitive impairment subjects, up to patients with Alzheimer’s disease, and that the metabolism of some acyl-carnitines is finely connected among them. These findings also suggest that the serum levels of acetyl-L-carnitine and other acyl-L-carnitines could help to identify the patients before the phenotype conversion to Alzheimer’s disease and the patients who would benefit from the treatment with acetyl-L-carnitine. However, further validation on a larger number of samples in a longitudinal study is needed before application to clinical practice.

## Introduction

Among the major challenges of modern research there are the treatment and prevention of Alzheimer's disease (AD), the most common and fearsome form of dementia. Researches, focusing on the characterization of the earliest, preclinical stages of dementia, identified transitional states between normal aging and AD, known as mild cognitive impairment (MCI) and subjective memory complaint (SMC). MCI refers to the clinical condition in which persons experience memory loss and/or other cognitive impairments greater than expected for age and level of education, but they do not fulfill accepted criteria for dementia. This condition is at high risk for developing AD and is suitable for possible therapeutic intervention [1, 2]. The term subjective memory complaint (SMC) is generally used to indicate a report of memory problems without pathological results on neuropsychological tests, which may or may not be perceived by others. However, there is no consensus on a standard definition for this symptom. There is growing evidence that SMC in the elderly, without organic or identifiable condition known to cause memory disturbance, is associated with an increased risk of cognitive decline and dementia [3, 4]. Some studies have shown structural, functional, and metabolic neuroimaging changes in SMC that closely resemble those seen in AD [5–7]. The subjects with MCI and/or SMC therefore should be identified and possibly treated to prevent the progression to dementia.

Acetyl-L-carnitine (ALC) is an endogenous molecule that is synthesized in the mitochondria by acetylation of L-carnitine. Approximately, the adult brain contains 80% of FC, 10–15% of ALC, and less than 10% long chain acyl-carnitines [8]. ALC has many different functions in the body and its supplementation has shown beneficial effects in the treatment of AD, producing a slower progression of mental deterioration [9–11]. However, larger multicenter, randomized controlled trial found no significant effect on cognitive decline [12, 13]. The authors of a recent meta-analysis [14] concluded that there is no sufficient evidence to recommend the routine use of ALC in clinical practice and that more studies, particularly on pharmacokinetics of ALC, are needed [14]. Although many studies have investigated the neurobiological properties of ALC and its supplementation effects in several neurological diseases including AD, the concentration of endogenous ALC and other acyl-carnitines in subjects with different severity of cognitive disturbances has yet to be fully investigated.

Aim of this study was to determine the endogenous levels of ALC and other acyl-L-carnitines in different groups of subjects along the continuum from normal to SMC, MCI, up to AD, and verify if such metabolites could be used as potential serum biomarkers of AD progression.

## Materials and Methods

### Study population

One hundred and seventeen participants were consecutively recruited from the Centre for Research and Training in Medicine for Aging (CeRMA), University of Molise. They were divided into four groups based on their clinical profiles: 29 participants with probable AD (7 males, 22 females, mean age±SD: 71.4±9.5 years), 18 with amnestic MCI (4 males, 14 females, mean age±SD: 69.4±8.2 years), 24 with SMC (8 males, 16 females, mean age±SD: 66.0±7.6 years) and 46 HS (20 males, 26 females, mean age±SD: 65.0±7.1 years). The characteristics of participants are summarized in Table 1. The body mass index (BMI) of AD patients was slightly lower than in other groups (Table 1), but the differences were not significant (F = 2.250; df = 3, 113; p = 0.086). The Mini Nutritional Assessment (MNA) [15] showed that all participants were well-nourished (MNA score > 23.5), with the exception of 11 AD patients who were at risk of malnutrition (MNA score between 18 and 23), but not malnourished.

**Table 1:** Demographic and clinical characteristics of study groups.

	Group			
	AD (n. 29)	MCI (n. 18)	SMC (n. 24)	HC (n. 46)
Age (mean+SD, y)	71.4+9.5	69.4+8.2	66.0+7.6	65.0+7.1
Male (n, %)	7 (5.9%)	4 (3.4%)	8 (6.8%)	20 (17.1%)
Female (n, %)	22 (18.8%)	14 (11.9%)	16(13.7%)	26 (22.2%)
Education level (mean+SD, y)	8.9+4.9	9.3+4.1	11.8+3.0	12.4+3.3
BMI (mean+SD, kg/m^2^)	25.8+5.4	27.78+3.6	27.52+4.1	28.56+4.1
MMSE (mean+SD, score)	12.6+8.0	26.7+2.2	29.6+0.7	29.6+0.7
Smoke^a^ (n, %)	8; 27.6%	5; 27.7%	5; 20.8%	12; 26.1%
Dyslipidemia (n, %)	10; 34.5%	5; 27.8%	6; 25.0%	12; 26.1%
Diabetes (n, %)	7; 24.1%	4; 22.2%	4; 16.7%	6; 13.0%
Hypertension (n, %)	14; 48.3%	9; 50.0%	10; 41.7%	18; 39.1%
Arrhythmia (n, %)	2; 6.9%	2; 11.1%	2; 8.3%	3; 6.5%
Heart failure^b^ (n, %)	2; 6.9%	-	-	2; 4.3%
TIA/Stroke (n, %)	2; 6.9%	1; 5.5%	2; 8.3%	1; 2.2%
Chronic kidney disease^c^ (n, %)	1; 3.4%	-	-	-
Prior Tumors (n, %)	3; 10.3%	1; 5.5%	3; 12.5%	5; 10.9%
Antihypertensive (n, %)	14; 48.3%	9; 50.0%	10; 41.7%	18; 39.1%
Lipid-lowering (n, %)	5; 17.2%	5; 27.8%	6; 25.0%	9; 19.6%
Hypoglycemic (n, %)	7; 24.1%	4; 22.2%	4; 16.7%	6; 13.0%
Antiacid (n, %)	8; 27.6%	3; 16.7%	6; 25.0%	8; 17.4%
Antiplatelet (n, %)	7; 24.1%	2; 11.1%	5; 20.8%	7; 12.2%
Supplements (n, %)	5; 17.2%	3; 16.7%	5; 20.8%	6; 13.0%

^a^, current or former smoker

^b^, subjects in NYHA (New York Heart Association) class I-II

^c^, subjects with glomerular filtration rate (GFR)>30 mL/min/1.73 m^2^

-, none; AD, Alzheimer disease; MCI, mild cognitive impairment; SMC, subjective memory complaint; HS, healthy subjects; BMI, Body mass index; MMSE, Mini Mental State Examination; TIA, transient ischemic attack.

Patients with probable AD were diagnosed according to National Institute of Neurological and Communicative Diseases and Stroke/Alzheimer’s Disease and Related Disorders Association (NINCDS-ADRDA) criteria [16] and presented Mini Mental State Examination (MMSE) score<24 [17] and Clinical Dementia Rating (CDR) score>0.5 [18]. Subjects with amnestic MCI met the Petersen’s diagnostic criteria [1], had MMSE≥24 and CDR = 0.5, and showed memory impairment as assessed via age-sex-education-adjusted scores on at least one of the following tests: Rey's word list immediate and delayed recall [19] and Prose memory, immediate and delayed [20]. Participants with SMC stated that their memory function has deteriorated compared to earlier stages in life, reported that the time of onset was in adulthood, had a score of 25 or more on the Memory Complaint Questionnaire (MAC-Q) [21] and showed normal objective memory performance on Rey's and Prose memory tests [3]. To summarize, MCI subjects showed both subjective and objective memory impairment, SMC participants presented only memory complaints with a normal score on the memory tests and HS showed neither subjective nor objective memory impairment. Depression at screening was assessed with the Geriatric Depression Scale (GDS) [22], and participants with a GDS score of 6 or more were considered depressed and excluded from the study. The patients on treatment with cerebro-active drugs underwent a wash-out period of at least 14 days before assessment. This study was conducted in accordance with ethical principles stated in the Declaration of Helsinki, as well as with approved national and international guidelines for human research. The Ethics Committee of University of Molise reviewed and approved this study, and subjects or caregivers were completely informed of the study procedures and provided written informed consent.

### Blood collection and biomarker analyses

Blood collection was done between 8:00 and 8:30 AM after an overnight fasting of at least 8–10 hours. Venous blood was collected into vacutainer serum tubes (Becton & Dickinson, Milan, Italy) and centrifuged within four hours. All serum samples were stored at -80°C until the preparation of spots and shipment to the analytical laboratory. To prepare spot serum from patients, 20 μl of each serum were spotted on filter paper (Whatman, 903, Gmbh, Dassel Germany), dried, and sent by courier to the laboratory at room temperature. The analytical laboratory was blinded to sample identification codes. Sample preparation from serum, preparation of internal quality controls and metabolite analyses were performed according to the conventional validated method as previously reported [23–25].

Briefly, labeled standards of acyl-carnitines were purchased from Cambridge Isotope Laboratories (Andover, MA, USA); a stock solution was made in methanol, and the standard concentrations were in the range 7.6–152 mmol/L for acyl-carnitines. In order to obtain working solutions, daily dilutions (1:100) were made using methanol. All chemicals and solvents were of the highest purity available from commercial sources, and were used without any further purification. To prepare serum calibrators, different amounts of acyl-carnitines standard solution were spiked into drug-free serum samples. Twenty μl of each spiked serum were spotted on filter paper (Whatman, 903, Gmbh, Dassel Germany) and let to dry. The dried serum spot was automatically punched (a disk of 3.2 mm of ∅ contains about 2 μL of serum) into a 1.5-mL tube, and 200 μL of methanol containing labeled standards were added. The sample was shaken on a vortex system for 20 min, thereafter was dried under a nitrogen flow at 50°C. The extracted acyl-carnitines were re-suspended using 200 μL of water/acetonitrile (1:1) containing 0.1% formic acid, and 40 μL of sample solution were injected using the flow injection analysis (FIA) mode into the mass spectrometer.

Targeted analysis of FC and acyl-carnitines was performed by tandem mass spectrometry (MS/MS). Samples were delivered directly into the TurbolonSpray ionization source of an API 4000 (SCIEX, Toronto, Canada) instrument. Labeled internal standards of FC and acyl-carnitines were purchased from Cambridge Isotope Laboratories (Andover, MA, USA); a stock solution was made in methanol. The standard concentrations were in the range of 7.6–152 μmol/L for FC and acyl-carnitines, the daily working solution was made by dilution of the stock solution (1:200) using methanol–water 90:10 v/v. The analytes concentrations were calculated automatically using the Chemoview software (SCIEX, Toronto, Canada). The ionization source operated under positive ion mode at a voltage of 5500 volts and with a turbo gas flow of 10 L/min of air, heated to 450°C (nominal heating-gun temperature). Mass calibration and resolution adjustments on the resolving quadrupoles were performed automatically using a PPG 2 x 10^−6^ mol/L solution introduced via the built-in infusion pump. Mass resolution was set on both quadrupoles at 0.7 amu at half height of mass-peak for all MS and MS/MS experiments. Collision-activated dissociation MS/MS was performed through the LINAC Q2 collision cell, operating with 10 mTor pressure of nitrogen as collision gas. Declustering potential, collision exit potential and collision energy were automatically optimized for FC and acyl-carnitines by the "quantitation optimization" option. The resulting declustering potential was +50V, optimal collision energy and collision exit potential were set at 26V and 20V, respectively. The targeted metabolite profiling was focused on the estimation of free L-carnitine (FC or C0), acetyl-L-carnitine (ALC or C2); propionyl-L-carnitine (C3); acrylyl-L-carnitine (C3:1); malonyl-L-carnitine (C3-DC); butyryl-L-carnitine (C4); methylmalonyl-L-carnitine (C4-DC); 3-hydroxybutyryl-L-carnitine (C4-OH); isovaleryl-L-carnitine (C5); glutaryl-L-carnitine (C5-DC); 3-hydroxyisovaleryl-L-carnitine (C5-OH); tiglyl-L-carnitine (C5:1); hexanoyl-L-carnitine (C6); hexenoyl-L-carnitine (C6:1); adipyl-L-carnitine (C6-DC); octanoyl-L-carnitine (C8); octenoyl-L-carnitine (C8:1); decanoyl-L-carnitine (C10); decenoyl-L-carnitine (C10:1); 3-hydroxydecanoyl-L-carnitine (C10-OH); dodecanoyl-L-carnitine (C12); dodecenoyl-L-carnitine (C12:1); 3-hydroxydodecanoyl-L-carnitine (C12-OH); myristoyl-L-carnitine (C14); tetradecenoyl-L-carnitine (C14:1); tetradecadienoyl-L-carnitine (C14:2); 3-hydroxytetradacanoyl-L-carnitine (C14-OH); palmitoyl-L-carnitine (C16); hexadecenoyl-L-carnitine(C16:1); 3-hydroxyhexadecanoyl-L-carnitine (C16-OH); 3-hydroxyhexadecenoyl-L-carnitine (C16:1-OH); stearoyl-L-carnitine (C18); oleyl-L-carnitine (C18:1); linoleyl-L-carnitine (C18:2); 3-hydroxyoctadecanoyl-L-carnitine (C18-OH); 3-hydroxyoctadecenoyl-L-carnitine (C18:1-OH).

Analytical variability (CV%) was estimated during the analysis of samples on three different levels (μmol/L) of quality controls of free L-carnitine and acyl-L-carnitines (S1 Table). In addition, we calculated 19 molar ratios as additional markers: C2/C0, C3/C0, C3/C4, C5/C3, C5/C4, C5/C8, C3/C16, C5DC/C4, C5DC/C8, C5DC/C12, C8/C6, C8/C6, C8/C10, C14:1/C16:1, C14:1/C5, C14:1/C4, C14:1/C8, C14/C14:1, C0/(C16+C18), (C16+C18:1)/C2.

### Statistical analysis

Data were analyzed using the SPSS (v. 17.0) statistical software package (SPSS Inc., Chicago, USA). Variables were examined for outliers and extreme values by means of box and normal quantile-quantile plots, and Shapiro-Wilk’s and Kolmogorov-Smirnov’s tests. When normal distribution could not be accepted, variable transformations (square, square root, logarithmic, reciprocal of square root or reciprocal transformations) were reviewed. The reciprocal of square root of FC, C2, C4-OH, C5, C5-DC, C5-OH, C8, C10, C10:1, C10-OH, C12, C12:1, C16, and C18:1, the logarithm of C3, C3:1, C3-DC, C4, C4-DC, C6-DC, C12-OH, C14, C14:1, C14:2, C14-OH, C18:2 and C18-OH and, finally, the natural logarithm of C6 and C6:1 concentrations helped to improve the distribution shape. Group differences (HS vs. SMC vs. MCI vs. AD) were evaluated by one-way multivariate analysis of variance (MANOVA) with age, gender, education level and body mass index (BMI) as covariates. The assumption of equality of variance was assessed by means of Levene's test. Finally, post-hoc pairwise multiple comparison using Bonferroni's correction was performed in order to detect significant differences among mean groups.

Multivariate statistical analysis was also performed using projection methods as implemented in MetaboAnalyst 2.0 on-line package [26, 27]. Principal component analysis (PCA) was first applied to detect sample metabolites trends and clusterings in an unsupervised manner, and the partial least-squares discriminant analysis (PLS-DA) was applied to reinforce classification and to better identify clusterings. The concentrations of all variables analyzed were preprocessed by normalization by the sum, transformed by Log normalization, and with mean-centered and auto-scaling before to built the model. The model quality was evaluated by the goodness-of-fit parameter (R2) and the goodness-of-prediction parameter (Q2). The classification performance of selected metabolites was assessed using the receiver operating characteristic (ROC) curve and the area under the curve (AUC).

## Results

MANOVA, including age, gender, education level and BMI as covariates, showed a statistically significant differences between groups (F = 2.005; df = 105, 231; p<0.001; Pillai's Trace = 1.430; partial η^2^ = 0.477). The results of univariate ANOVA and post hoc multiple comparisons are reported in Table 2. Endogenous concentrations of ALC progressively decreased from HS (mean±SD, 5.547±1.308 μmol/L) to SMC (4.343±0.869 μmol/L), from SMC to MCI (4.013±0.526 μmol/L) and from MCI to AD (3.529±0.639 μmol/L) group [F = 24.589; df = 3, 109; p<0.001; partial η^2^ = 0.404] (Table 2). Pairwise multiple comparisons showed that HS was significantly different from SMC (p<0.005), MCI (p<0.005) and AD (p<0.005) group, and this latter was significantly different from SMC (p<0.005) group. The comparisons SMC vs. MCI (p = 1.000) and AD vs. MCI (p = 0.082) were not significant (Table 2). Other acyl-carnitines, such as C3-DC, C5-OH, C6:1, C10, C12, C12:1, C14, C14:1, C16:1, C18, C18:1 and C18:2 showed a similar behavior, decreasing from HS through SMC and MCI up to AD patients (Table 2). However, while the comparison HS vs. AD was significant for all such acyl-carnitines, the comparison MCI vs. HS was also significant for C10, C12:1, C14:1, C16:1, C18 and C18:1, and the comparison HS vs. SMC for C16:1 and C18. For the acyl-carnitines C3, C8 and C10:1, only the comparison HS vs. MCI was significant. FC did not show significant differences between groups (Table 2).

**Table 2:** Serum levels (μmol/L) of free L-carnitine and acyl-carnitines in the four studied groups.

Metabolites	Group*				ANOVA		Significant pairwise comparisons
	AD	MCI	SMC	HS	*F* (3, 109)	p value	
**Free L-carnitine (C0)**	28.302 (5.911)	27.974 (5.226)	26.969 (4.141)	28.923 (6.301)	0.387	0.763	n.a.
**Acetyl- (C2)**	3.529 (0.639)	4.013 (0.526)	4.343 (0.869)	5.547 (1.308)	24.589	<0.001	AD vs SMC; AD vs HS; MCI vs HS; SMC vs HS
**Propionyl- (C3)**	0.182 (0.076)	0.149 (0.052)	0.175 (0.048)	0.215 (0.075)	4.187	0.008	MCI vs HS
**Acrylyl- (C3:1)**	0.016 (0.009)	0.017 (0.007)	0.015 (0.006)	0.013 (0.005)	0.931	0.428	n.a.
**Malonyl- (C3-DC)**	0.026 (0.007)	0.028 (0.009)	0.032 (0.012)	0.036 (0.014)	4.823	0.003	AD vs HS
**Butyryl- (C4)**	0.169 (0.097)	0.182 (0.219)	0.144 (0.052)	0.156 (0.076)	0.160	0.923	n.a.
**Methylmalonyl- (C4-DC)**	0.039 (0.015)	0.038 (0.011)	0.035 (0.012)	0.040 (0.015)	0.814	0.489	n.a.
**3-hydroxybutyryl- (C4-OH)**	0.025 (0.008)	0.026 (0.007)	0.023 (0.008)	0.027 (0.009)	2.584	0.057	n.a.
**Isovaleryl- (C5)**	0.071 (0.031)	0.075 (0.023)	0.073 (0.022)	0.082 (0.028)	1.409	0.244	n.a.
**Glutaryl- (C5-DC)**	0.032 (0.012)	0.029 (0.008)	0.040 (0.017)	0.040 (0.016)	3.117	0.029	n.s.
**3-hydroxyisovaleryl- (C5-OH)**	0.037 (0.010)	0.043 (0.010)	0.041 (0.010)	0.045 (0.011)	3.217	0.026	AD vs HS
**Tiglyl- (C5:1)**	0.033 (0.006)	0.033 (0.009)	0.036 (0.009)	0.034 (0.008)	0.868	0.460	n.a.
**Hexanoyl- (C6)**	0.042 (0.017)	0.038 (0.009)	0.036 (0.011)	0.047 (0.036)	1.289	0.282	n.a.
**Hexenoyl- (C6:1)**	0.066 (0.014)	0.069 (0.015)	0.072 (0.020)	0.081 (0.018)	3.776	0.013	AD vs HS
**Adipyl- (C6-DC)**	0.035 (0.014)	0.035 (0.013)	0.034 (0.009)	0.034 (0.012)	0.004	1.000	n.a.
**Octanoyl- (C8)**	0.126 (0.047)	0.115 (0.034)	0.135 (0.047)	0.160 (0.133)	3.177	0.027	MCI vs HS
**Octenoyl- (C8:1)**	0.063 (0.019)	0.065 (0.020)	0.069 (0.022)	0.070 (0.024)	0.696	0.557	n.a.
**Decanoyl- (C10)**	0.117 (0.079)	0.104 (0.043)	0.136 (0.080)	0.161 (0.159)	5.694	0.001	AD vs HS; MCI vs HS
**Decenoyl- (C10:1)**	0.079 (0.041)	0.063 (0.019)	0.079 (0.034)	0.089 (0.047)	3.643	0.015	MCI vs HS
**3-hydroxydecanoyl- (C10-OH)**	0.032 (0.012)	0.029 (0.008)	0.040 (0.017)	0.040 (0.016)	3.117	0.029	n.s.
**Dodecanoyl- (C12)**	0.043 (0.020)	0.052 (0.023)	0.054 (0.021)	0.072 (0.064)	7.322	<0.001	AD vs SMC; AD vs HS
**Dodecenoyl- (C12:1)**	0.046 (0.022)	0.044 (0.018)	0.052 (0.017)	0.061 (0.019)	7.297	<0.001	AD vs HS; MCI vs HS
**3-hydroxydodecanoyl- (C12-OH)**	0.015 (0.005)	0.014 (0.006)	0.016 (0.007)	0.017 (0.006)	1.480	0.224	n.a.
**Myristoyl- (C14)**	0.029 (0.011)	0.033 (0.008)	0.031 (0.009)	0.039 (0.016)	4.293	0.007	AD vs HS
**Tetradecenoyl- (C14:1)**	0.045 (0.025)	0.041 (0.015)	0.048 (0.022)	0.056 (0.026)	4.169	0.008	AD vs HS; MCI vs HS
**Tetradecadienoyl- (C14:2)**	0.039 (0.015)	0.042 (0.018)	0.035 (0.014)	0.038 (0.011)	0.798	0.498	n.a.
**3-hydroxytetradecanoyl- (C14-OH)**	0.010 (0.005)	0.008 (0.003)	0.009 (0.003)	0.009 (0.003)	1.699	0.172	n.a.
**Palmitoyl- (C16)**	0.101 (0.043)	0.165 (0.085)	0.132 (0.057)	0.120 (0.062)	3.548	0.017	AD vs MCI
**Hexadecenoyl- (C16:1)**	0.027 (0.008)	0.027 (0.007)	0.025 (0.005)	0.034 (0.010)	7.462	<0.001	AD vs HS; SMC vs HS; MCI vs HS
**3-hydroxyhexadecanoyl- (C16-OH)**	0.007 (0.003)	0.007 (0.002)	0.007 (0.003)	0.007 (0.004)	0.440	0.725	n.a.
**3-hydroxyhexadecenoyl- (C16:1-OH)**	0.008 (0.003)	0.006 (0.003)	0.006 (0.004)	0.007 (0.003)	1.308	0.276	n.a.
**Stearoyl- (C18)**	0.043 (0.011)	0.044 (0.011)	0.045 (0.011)	0.056 (0.016)	5.275	0.002	AD vs HS, SMC vs HS, MCI vs HS
**Oleyl- (C18:1)**	0.105 (0.031)	0.109 (0.025)	0.116 (0.031)	0.135 (0.039)	5.016	0.003	AD vs HS; MCI vs HS
**Linoleyl- (C18:2)**	0.033 (0.008)	0.036 (0.009)	0.038 (0.010)	0.042 (0.012)	2.700	0.049	AD vs HS
**3-hydroxyoctadecanoyl- (C18-OH)**	0.007 (0.004)	0.007 (0.003)	0.006 (0.003)	0.009 (0.004)	3.004	0.034	SMC vs HS
**3-hydroxyoctadecenoyl- (C18:1-OH)**	0.009 (0.004)	0.007 (0.003)	0.007 (0.003)	0.009 (0.003)	2.842	0.041	n.s.

AD, Alzheimer disease; MCI, mild cognitive impairment; SMC, subjective memory complaint; HS, healthy subjects; n.a., not applicable; n.s., not significant; ^*^ data are means (standard deviation).

**Fig 1:**
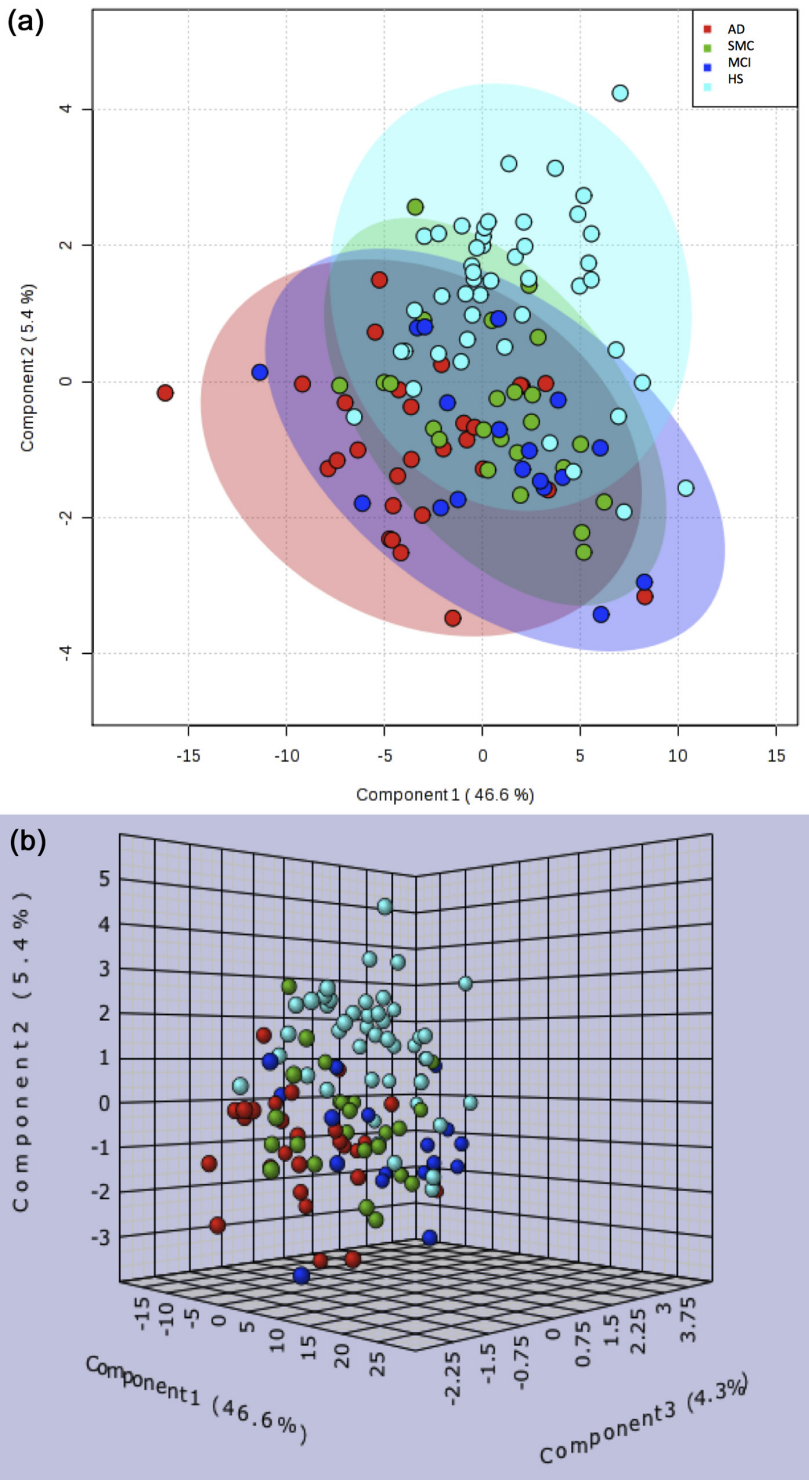
Score plots for the PLS-DA model discriminating the 4 groups of subjects. Plots of the first two (A) and first three (B) components that explain, respectively, the 52% and the 56.3% of model variance. AD (red) samples were well separated from HS (light blue), while MCI (blue) and SMC (green) clustered in an intermediate zone.

The projections of PCA are reported in S1A Fig. The first three principal components, obtained from the original data set, explained the 62% of data variance. The PCA score in the new coordinate system allowed a good separation of AD samples from samples of the other subjects (MCI, SMC and HS). PLS-DA (S1B Fig) showed a better separation between AD and HS, while both MCI and SMC classes clustered very closed and in an intermediate zone. The first two components of PLS-DA explained the 52% of the model, and both MCI and SMC were distributed between AD patients and HS (Fig 1A). The Fig 1B shows a 3D score plot of PLS-DA, in which the combination of the first three components clearly separated AD samples (red) from HS (light blue), while MCI (blue) and SMC (green) clustered in an intermediate zone.

The panel A of S2 Fig shows the first three latent variables of PLS-DA used to build the model: the prediction accuracy (Accuracy) based on cross-validation, the sum of squares captured by the model (R2), and the cross-validated R2 (Q2). The optimal number of components (n = 3) was determined by Q2 (*). To avoid the propensity of PLS-DA to data overfitting, a permutation test was performed to detect it. The panel B of S2 Fig shows the result of permutation test, which randomly reassigned the class labels and performed PLS-DA on the newly relabeled data set. The process was repeated thousand time, and the test was statistically significant (p<0.001, less than 1/1,000).

**Fig 2:**
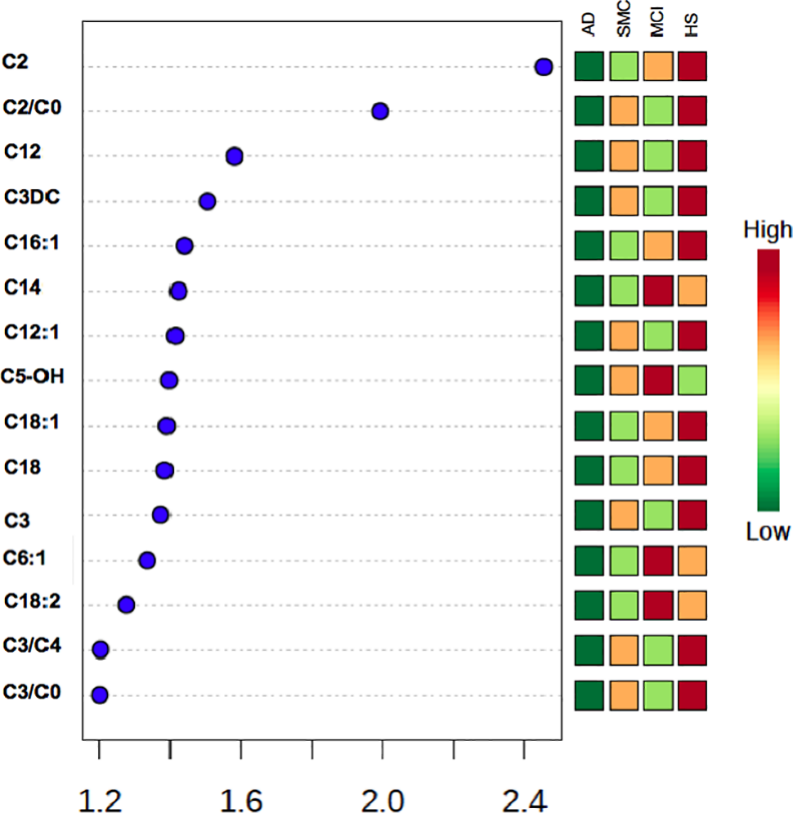
Important features identified by PLS-DA. Twelve metabolites and 3 molar ratios show a VIP score > 1.3.The colored boxes indicate the relative concentrations of the corresponding metabolite or the relative value of ratios in each group.

**Fig 3:**
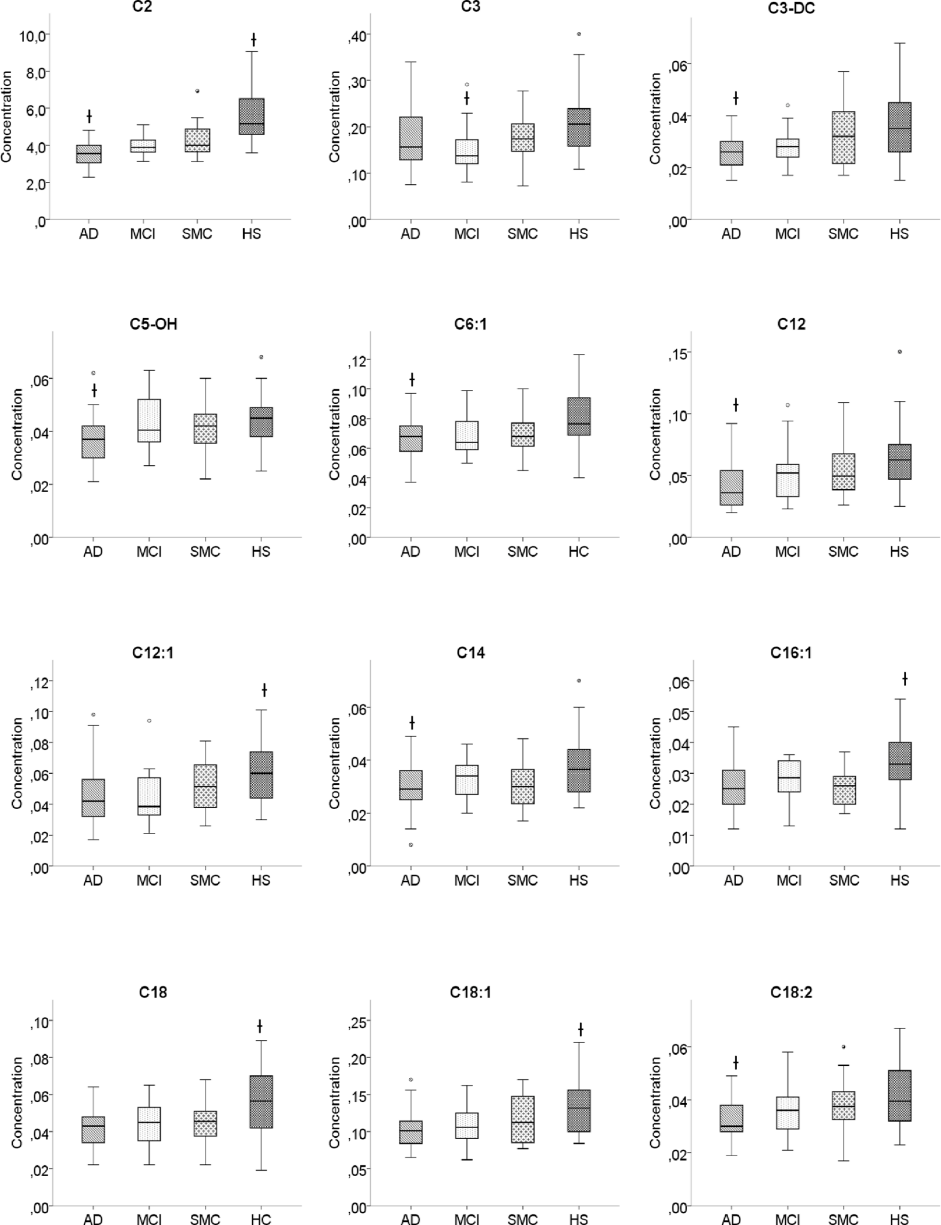
Serum levels of acyl-carnitines classified as VIP by PLS-DA. Box-plots show median (horizontal line in the box), 25th and 75th percentiles (edges of box), maximum and minimum values (whiskers) and outliers (°,*) of acyl-carnitines concentrations (μmol/L) in the 4 groups of subjects. AD, Alzheimer’s disease; MCI, mild cognitive impairment; SMC subjective memory complaint; HS, healthy subjects; (†) Significantly different from HS groups (see Table 2 for details).

**Fig 4:**
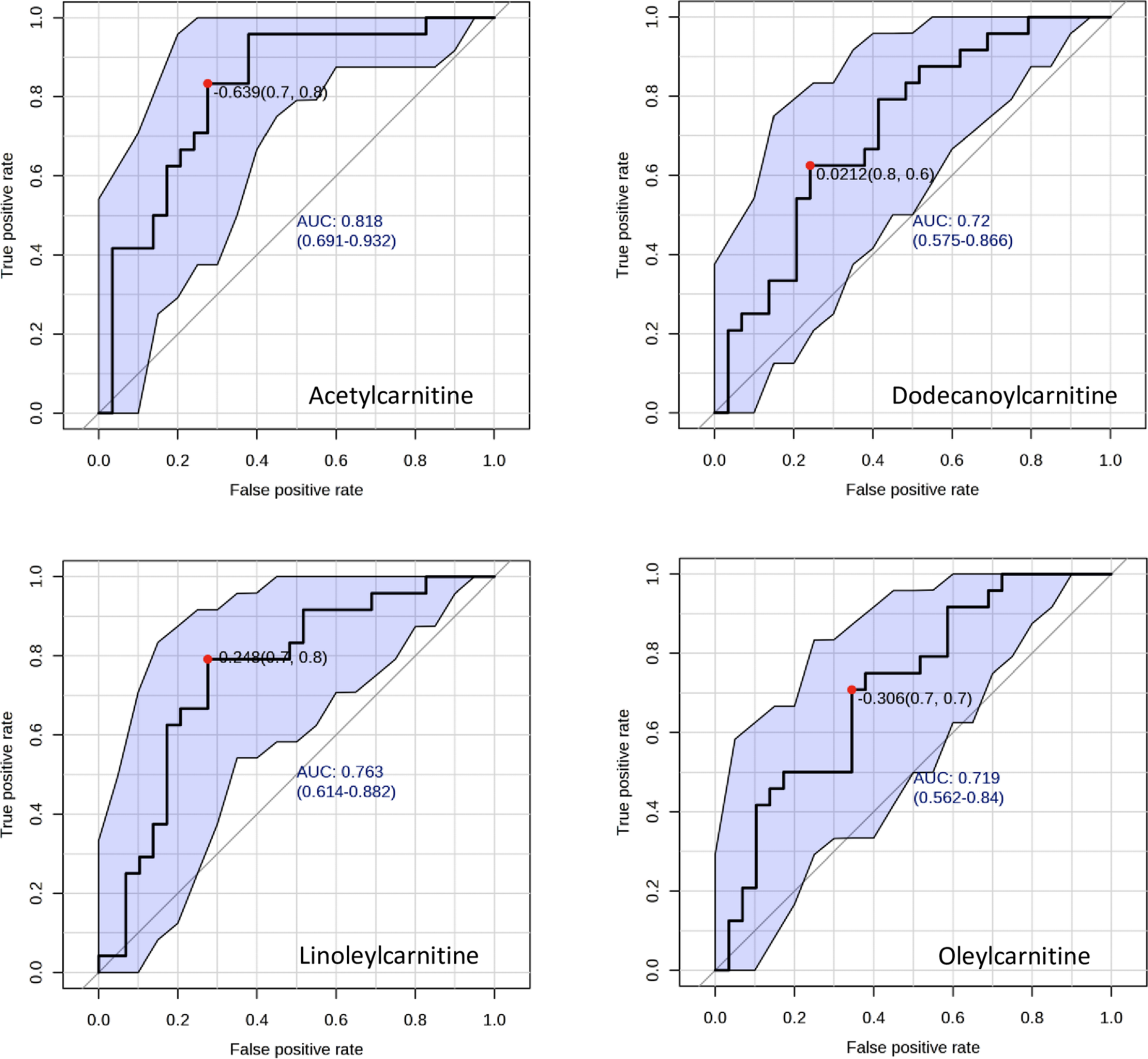
ROC curves of selected acyl-carnitines. The plots show the optimal value of cutoff (•), the value of full AUC with the 95% confidence intervals and the best delimitation of AUC (black solid line) for each metabolite.

The 12 metabolites (C2, C12, C3DC, C16:1, C14:0, C12:1, C5-OH, C18:1, C18:0, C3, C6:1 and C18:2) and the 3 molar ratios (C2/C0, C3/C4 and C3/C0), identified by PLS-DA as VIP (variable importance in projection) with a score >1.3, are reported in Fig 2. Fig 3 reports the box-plots of 12 acyl-carnitines classified as VIP from PLS-DA.ROC analysis showed that C2, C12, C18:1 and C18:2, identified as significant features by both PLS-DA and ANOVA, yielded an AUC ranging from 0.82 (C2) to 0.72 (C12 and C18:1) (Fig 4).

## Discussion

To the best of our knowledge, this research is the first to investigate the serum levels of endogenous acyl-carnitines in different groups of subjects along the continuum from normal to AD, including subjects with SMC. Our results show a progressive decrease of ALC and other acyl-carnitine serum levels passing from HS through SMC and MCI up to AD groups (Table 2). Post hoc pairwise comparisons showed that 3 acyl-carnitines (C2, C16:1 and C18) significantly decreased in AD, MCI and SMC, 4 acyl-carnitines (C10, C12:1, C14:1 and C18:1) in AD and MCI, and 6 (C3-DC, C5-OH, C6:1, C12, C14 and C18:2) only in AD, compared to HS. Three acyl-carnitines (C3, C8 and C10:1) were significantly reduced in MCI compared to HS (Table 2). In particular, ALC significantly decreased on average by 21% in SMC, 27% in MCI and 36% in AD, compared to HS. No metabolites changed significantly between SMC and MCI, and FC did not show significant changes among groups (Table 2). Twelve metabolites and 3 molar ratios were identified by PLS-DA as VIP with a score > 1.3 (Fig 3). ROC curves (Fig 4) showed that the diagnostic accuracy of ALC was very good (AUC = 0.82) and that of other acyl-carnitines, such as C12, C18:1 and C18:2, was good (AUC between 0.7 and 0.8), suggesting that these metabolites could be proposed as potential biomarkers for the diagnosis of AD.

Previous studies, evaluating the serum level of FC and several acyl-carnitines in subjects with cognitive complaints, studied patients with AD or MCI [28, 29] or converters to MCI/AD [28], but not subjects with SMC. These studies were performed by different methodological approaches, therefore it is difficult to compare these results with that obtained in our study. Mapstone et al. [28] used an ultraperformance liquid chromatography–electrospray ionization quadrupole time-of-flight mass spectrometry (UPLC-ESI-QTOF-MS)-based data acquisition, and least absolute shrinkage and selection operator (LASSO) analysis for untargeted metabolomic profiling. They reported significant low levels of some acyl-carnitines such as C3 and C16:1-OH in the converter group, before and after phenoconversion to MCI/AD, and in the MCI/AD group. González-Domínguez et al. [29] used a capillary electrophoresis–mass spectrometry (CE-MS) approach for signal acquisition and a complex data processing, including a PLS-DA analysis, for untargeted metabolomic profiling. They found in patients with AD and in subjects with MCI a significant decrease of FC and C5, and a significant increase of C8, C10 and C10:1. In older studies, Rubio et al. [30] reported significantly lower levels of plasma FC in AD patients, while plasma acyl-carnitines and cerebrospinal fluid FC and acyl-carnitines were within normal range. Makar et al. [31] reported reduced concentration of “total” carnitine (FC plus acid-soluble carnitine esters) in several brain regions of AD patients, but such reduction was not significant. A part from the diversity of methodological approaches, other possible explanations of discrepant results might be the different number and characteristic of examined subjects, and the complex, multifactorial and multi-genetic nature of AD. Anyway, supporting our findings, González-Domínguez et al. [32] demonstrated the deficit of several acyl-carnitines, including ALC, in the brain (hippocampus and cerebral cortex) of APP/PS1 transgenic mouse model of AD.

The deficit of acyl-carnitines suggests a perturbed transport of fatty acids into the mitochondria for beta-oxidation, as well as an altered energy metabolism. This hypothesis is in line with the results of transcriptomic study showing a significant decrease in the activity of carnitine shuttle in AD patients [33]. The deficit of carnitine shuttle might contribute to mitochondrial dysfunctions supposed to be responsible for many neurodegenerative diseases including AD [34]. The decreased serum levels of some acyl-carnitines found in MCI subjects might indirectly signal an impending progression of dementia and might be used as biomarkers of phenotype conversion from MCI to AD.

ALC is a small, water-soluble and easily transportable compound, found naturally in the body and in small amounts in some foods. The synthesis of ALC and other acyl-carnitines occurs inside the mitochondria from acylation of free hydroxyl group of L-carnitine (trimethylamino-β-hydroxybutyrate) by the enzymes carnitine-acyltransferases, which catalyzes the exchange of acyl groups between carnitine and coenzyme A. These enzymes include carnitine acetyltransferase (CAT), carnitine octanoyltransferase (COT), and carnitine palmitoyltransferase (CPT) I and II, with substrate preferences for short chain, medium chain and long chain, respectively [35]. CPT-I (located in the inner side of the outer mitochondrial membrane) and CPT-II (in the inner leaflet of the inner mitochondrial matrix) are crucial for the β-oxidation of fatty acids in the mitochondria, by facilitating their transport across the mitochondrial membranes [36, 37]. CAT may be involved in the transport of acetyl-CoA across intracellular membranes, in maintaining the acetyl-CoA:CoA balance, and in the excretion of excess or harmful acyl molecules as acyl-carnitines [37, 38].

ALC has many different functions in the body. Because of its ability to go through the brain-blood-barrier freely, it could help fatty acid to enter into mitochondria and improve the brain’s energy metabolism. In addition, ALC could facilitate elimination of oxidative products, provides acetyl groups for acetylcholine synthesis, stimulates membrane phospholipid synthesis, exerts anti-apoptotic functions, modulates gene expression and neurotrophic factors production, and protects from excitotoxicity [8, 39–42]. All such functions would explain why its reduced levels may predispose to AD and may contribute to neurodegeneration underlining the disease.

ALC may be of benefit in treating several neurological disorders, including AD, depression in the elderly, HIV infection, diabetic neuropathies, peripheral neuropathic pain, brain ischemia, and cognitive impairment due to alcoholism [8, 43, 44]. However, despite its use in clinical studies, little is known about its metabolism in normal aging and in subjects with different degree of cognitive complaints. Kalaria and Harik [45] found a 25% to 40% decrease in CAT activity in several brain regions and in cerebral microvessels of patients with AD. Makar et al. [31] similarly reported a significant reduction of CAT activity in the cerebellum of AD patients. Based on the essential role of carnitine to transport fatty acids across the inner mitochondrial membrane for fatty acid oxidation, it is conceivable that the significant and progressive decrease of either short-, medium- and long-chain acylcarnitines in SMC, MCI and AD compared to HS may reflect a reduction in the activity of CAT, COT and CPT. It is likely that the deficiency of these enzymes, in particular COT and CPT I, could have a significant effect on energy production and could impair the mitochondrial functions. However, Perry et al. [46] described normal CAT activity in amygdaloid and caudate nuclei of AD patients, and Maurer et al. [47] reported normal activity of CPT, COT and CAT in several AD brain regions. Once again, differences in methods and examined populations, and the complexity of AD might explain the discrepant results. In effect, increasing evidences suggest that AD is a heterogeneous disorder that includes several subtypes with different pathogenic mechanisms and progression [48, 49].

Anyway, several studies suggested a beneficial effect of ALC on cognition and behavior in aging and AD subjects [9–11]. The possible mechanisms of ALC action in AD may involve restoring of cell membranes, as well as synaptic function, enhancing cholinergic activity, restoring brain energy, protecting against toxins, and exerting neurotrophic effects via stimulating NGF and acetylation of proteins [8, 39, 40]. However, later, larger studies have not supported these findings [12, 13]. In a recent meta-analysis including sixteen trials, Hudson and Tabet [14] noted that early and later studies differed widely in methodology and assessment tools used, and were therefore difficult to compare [14]. Anyway, the results of meta-analysis showed a statistically significant treatment effects in favor of ALC for clinical global impression at 12 and 24 weeks, and for MMSE at 24 weeks [14]. Similarly, another meta-analysis found significant treatment effects of ALC for clinical global impression and for several psychometric tests [50].

## Conclusion

The results of this study suggest that serum ALC and other acyl-carnitine concentrations decrease along the continuum from HS through SMC and MCI subjects, up to patients with AD, and that the metabolism of some acyl-carnitines is finely connected among them. Furthermore, the reduced level of ALC in subjects with cognitive complaints could explain the reported salutary effect of ALC administration in patients with AD. We suggest that the serum levels of ALC and other acyl-carnitines could help to identify the patients before the phenotype conversion to AD and the patients who would benefit from the treatment with ALC. However, further validation on a larger number of samples in a longitudinal study is needed before application to clinical practice.

## Supporting Information

S1 Fig **Score plots of PCA (panel A) and PLS-DA (panel B) analyzes.** PCA (panel A) of first three components shows a separation between AD (red triangle) and HS (light blue diamond). PLS-DA (panel B), a supervised classification method, maximizes the separation among the four groups of subjects.
(PDF)

S2 Fig **Prediction accuracy (panel A) and permutation test (panel B).** The panel A shows the prediction accuracy based on R2 and Q2 of the model (panel A). The best number of components (n = 3) to explain the results has been determined on the optimal value of Q2. The panel B shows the significance of permutation test based on thousand permuted class assignments.
(PDF)

S1 Table Analytical variability (CV%) of free L-carnitine and acyl-carnitines estimated on three different concentrations (μmol/L) of quality controls. (PDF)
