# Supplementary figures and images for: Serum Levels of Acyl-Carnitines along the Continuum from Normal to Alzheimer's Dementia
Source: PLoS One. 2016 May 19;11(5):e0155694. doi: 10.1371/journal.pone.0155694 (PMC4873244; doi:10.1371/journal.pone.0155694)

S1 Fig. Score plots of PCA (panel A) and PLS-DA (panel B) analyzes.

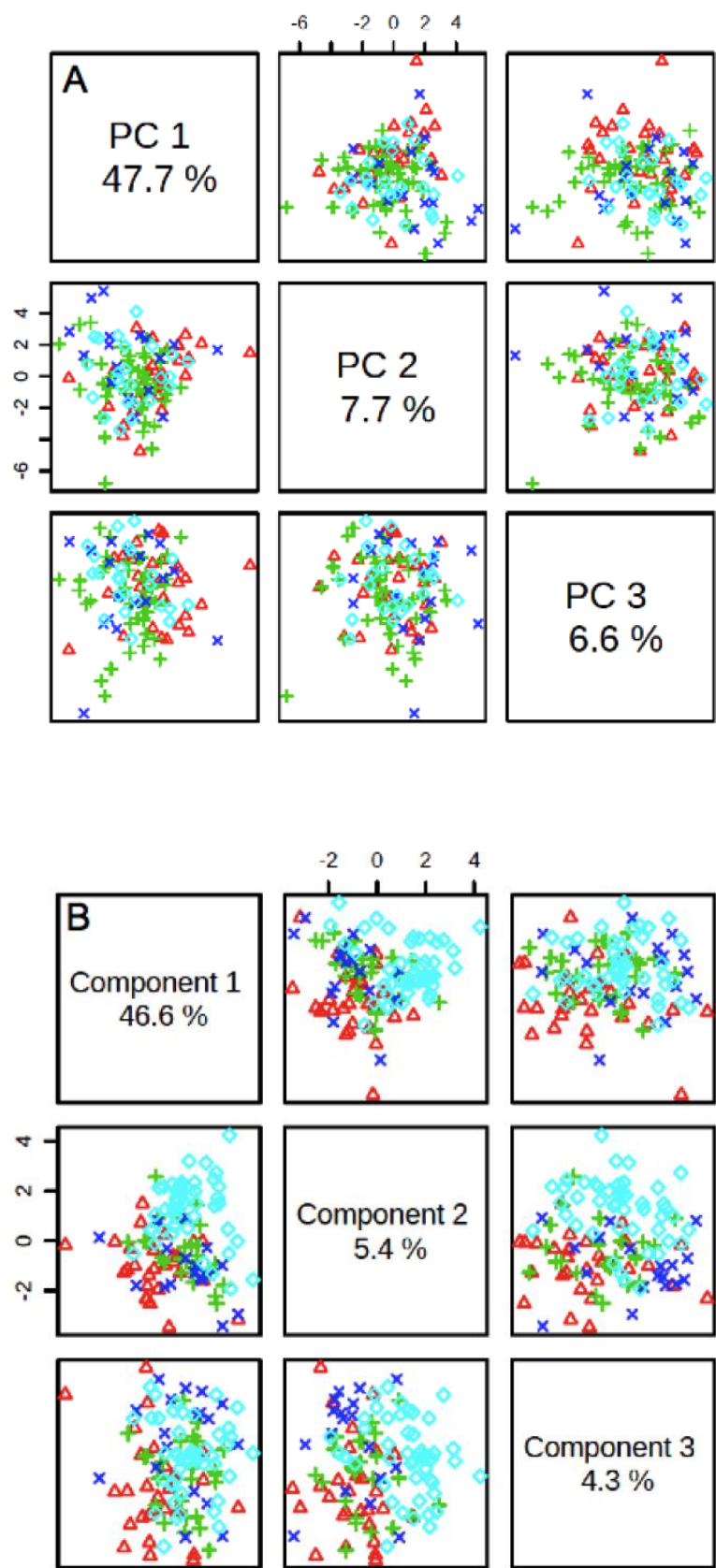

Supplement: S1 Fig — Score plots of PCA (panel A) and PLS-DA (panel B) analyzes. PCA (panel A) of first three components shows a separation between AD (red triangle) and HS (light blue diamond). PLS-DA (panel B), a supervised classification method, maximizes the separation among the four groups of subjects. (PDF) [file pone.0155694.s001.pdf]

S2 Fig. Prediction accuracy (panel A) and permutation test (panel B).

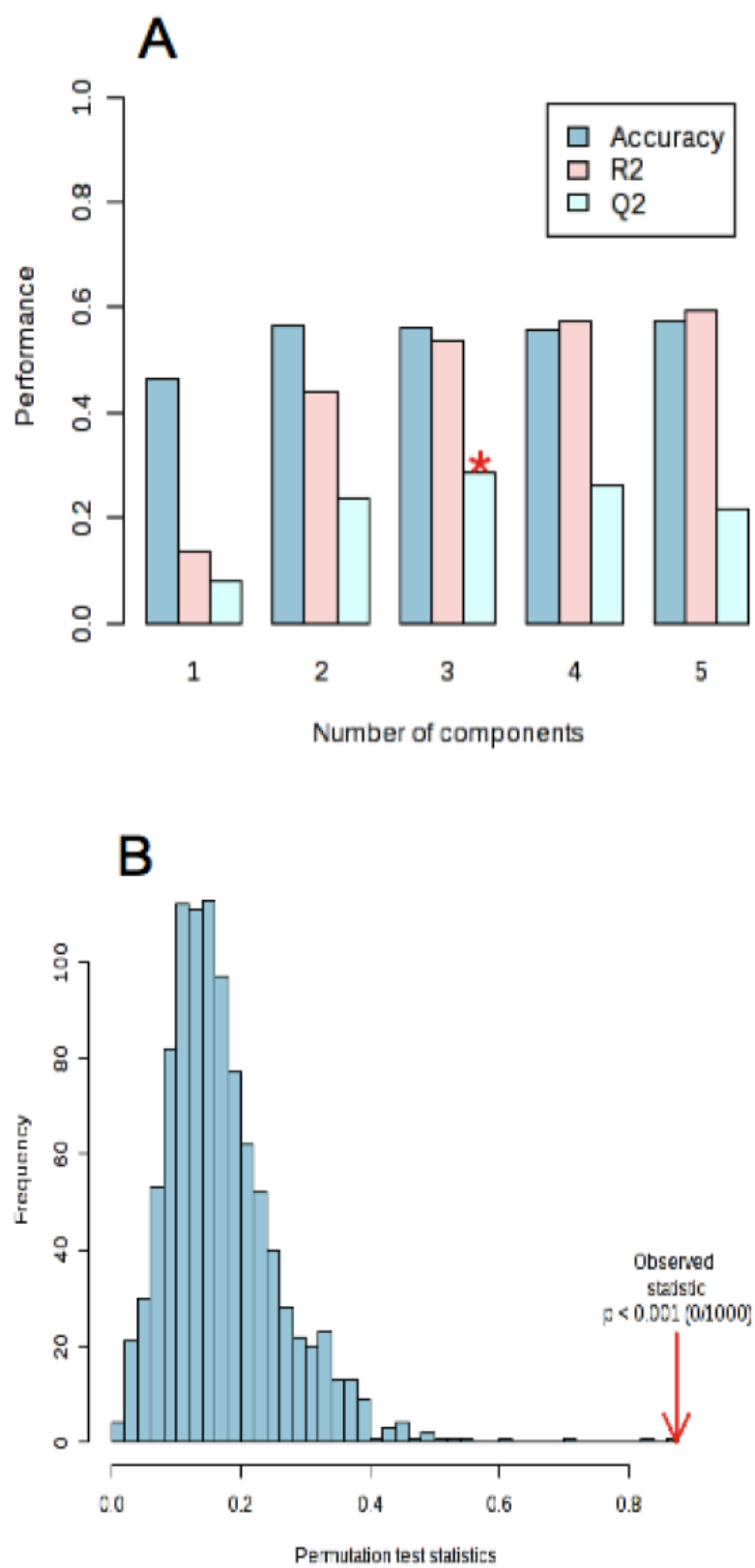

Supplement: S2 Fig — Prediction accuracy (panel A) and permutation test (panel B). The panel A shows the prediction accuracy based on R2 and Q2 of the model (panel A). The best number of components (n = 3) to explain the results has been determined on the optimal value of Q2. The panel B shows the significance of permutation test based on thousand permuted class assignments. (PDF) [file pone.0155694.s002.pdf]
