# Supplementary material for: Serum Levels of Acyl-Carnitines along the Continuum from Normal to Alzheimer's Dementia
Source: PLoS One. 2016 May 19;11(5):e0155694. doi: 10.1371/journal.pone.0155694 (PMC4873244; doi:10.1371/journal.pone.0155694)
Supplement: S1 Table — (PDF) [file pone.0155694.s003.pdf]

S1 Table. Tandem Mass Spectrometry variability (CV%) of free L-carnitine and acyl-carnitines estimated on three different concentrations (µmol/L) of quality controls.

	Level-1		Level-2		Level-3	
**Marker**	**mean**	**CV%**	**mean**	**CV%**	**mean**	**CV%**
**C0**	19.199	9.6	42.538	9.8	65.855	8.8
**C2**	9.424	16.5	14.178	10.6	39.035	10.3
**C3**	0.907	16.9	1.231	7.4	12.515	10.8
**C3DC**	0.059	13.6	0.079	14.8	2.002	10.7
**C4:0**	0.109	8.2	0.155	12.3	4.476	11.5
**C4DC**	0.500	14.3	0.590	11.5	2.037	15.4
**C4OH**	0.057	12.3	0.076	13.0	1.882	9.2
**C5:0**	0.067	10.0	0.102	15.5	2.576	9.1
**C5:1**	0.016	32.0	0.021	28.1	0.020	20.2
**C5DC**	0.015	15.6	0.029	22.4	0.735	9.4
**C5OH**	0.459	12.7	0.529	9.9	1.847	11.0
**C6:0**	0.020	10.8	0.029	15.2	1.885	10.2
**C8:0**	0.023	13.3	0.066	15.5	2.592	7.0
**C10:0**	0.035	34.0	0.091	18.7	2.337	12.2
**C10:1**	0.027	52.1	0.079	21.3	0.032	42.7
**C12:0**	0.017	25.0	0.034	15.2	1.619	9.0
**C12:1**	0.009	53.2	0.024	21.3	0.011	26.0
**C12-OH**	0.024	22.2	0.028	23.3	0.022	18.4
**C14:0**	0.058	9.3	0.085	16.2	2.834	7.7
**C14:1**	0.023	20.7	0.045	15.8	0.028	14.4
**C14:2**	0.011	23.7	0.023	17.9	0.013	20.4
**C14-OH**	0.012	52.4	0.019	55.7	0.022	28.0
**C16:0**	0.643	15.8	0.751	7.7	9.441	5.2
**C16:1**	0.034	32.6	0.050	12.1	0.038	27.0
**C16-OH**	0.020	9.6	0.038	16.4	0.667	12.2
**C16:1 OH**	0.038	10.1	0.068	15.4	0.054	9.0

**C18:0**	0.591	10.6	0.718	17.5	5.060	15.1
**C18:1**	0.869	21.6	1.126	17.1	1.002	18.6
**C18:2**	0.249	25.1	0.336	12.1	0.289	22.4
**C18-OH**	0.011	21.6	0.020	20.1	0.986	22.1
**C18:1-OH**	0.018	18.0	0.028	18.2	0.021	16.5
**C18:2 OH**	0.023	22.5	0.034	25.2	0.024	20.8
